# Method for Identifying Seismokinetic Effects in Earth’s Magnetic Field Measurements

**DOI:** 10.3390/s26134239

**Published:** 2026-07-03

**Authors:** Ivan Vassilyev, Vladimir Saveliev, Zhassulan Mendakulov, Vadim Lutsenko, Linara Zhadigerova, Andrey Malimbayev, Botakoz Seifullina, Jelena Caiko

**Affiliations:** 1 Special Design and Technology Bureau “Granit”, 292 Hussainov Street, Almaty 050060, Kazakhstan; iv@granit.kz; 2 Institute of Ionosphere, 117 “Ionosphere” Gardening Association, Kamenskoye Plateau, Almaty 050020, Kazakhstan; vladimir.l.saveliev@gmail.com (V.S.); vadim.lutsenko.y@gmail.com (V.L.); zhadigerova.l@ionos.kz (L.Z.); andrey.malimbayev@ionos.kz (A.M.); botanaika.93@gmail.com (B.S.); 3 Faculty of Computer Science, Information Technology and Energy, Riga Technical University, 12/1 Azenes Street, LV-1048 Riga, Latvia; jelena.caiko@gmail.com; 4 Natural Sciences and Information Technology Department, Riga Nordic University, 1 k-5 Valerijas Seiles Street, LV-1019 Riga, Latvia

**Keywords:** earthquake, inclinometer, magnetometer, magnetic observatory, Earth’s magnetic field, direction finding, correlation, high-frequency noise

## Abstract

Analysis of variations in the components of the Earth’s magnetic field, recorded at the magnetic observatory of the Institute of Ionosphere (Almaty, Kazakhstan) during the earthquakes of 22 January 2024 and 4 March 2024, showed that these fluctuations were not related to intrinsic variations in the magnetic field itself, but rather to mechanical oscillations of the magnetometer together with its support. Evidence supporting the seismokinetic origin of the variations in the vector magnetometer was the discrepancy between the total magnetic field *measured* by the absolute magnetometer and the total magnetic field *calculated* from the vector magnetometer readings. Equations were derived that relate variations in the components of the magnetic field to the tilt angles of the sensor, enabling the determination of the direction of arrival of seismic shock waves. Azimuths of earthquake epicenters were calculated from oscillations observed in magnetogram records for several events, and these were compared with azimuths obtained from a network of seismic stations. It is shown that, in order to improve the accuracy of geomagnetic field measurements—required for the study of seismoelectric and seismomagnetic phenomena, as well as for identifying earthquake precursors—the design of magnetic observatories should be supplemented with an inclinometer to enable correction of vector magnetometer measurement results.

## 1. Introduction

The study of the Earth’s magnetic field is one of the most important areas of scientific research. About 200 years ago, the first magnetic observatories were established to carry out regular observations of the magnetic field and its variations. These observations contribute to understanding the origin of the Earth’s magnetic field and its evolution, as well as the internal structure of the Earth. One of the key directions in studying the Earth’s magnetic field is the identification of variations associated with earthquakes and their preparation (i.e., the detection of earthquake precursors).

Earthquakes are among the most hazardous natural phenomena. Each year 250–300 destructive earthquakes occur, causing significant economic damage [1] and, more importantly, leading to loss of human life. Up to 40% of the total damage is associated not with the direct effects of seismic shaking, but with secondary factors related to earthquakes (such as tsunamis, landslides, epidemics, etc.) [2]. Unfortunately, humanity has not yet learned to predict earthquakes with the desired accuracy. Nevertheless, there is hope that this may become possible in the future, when scientists accumulate sufficient objective data on various physical phenomena accompanying the preparation of earthquakes that may serve as potential precursors. To address this challenge, the number of seismic stations worldwide is increasing, forming various national and international seismic monitoring networks [3,4]. The Japanese Hi-net observation network alone includes about 800 seismic stations [5].

Hypotheses about a possible influence of seismic activity on the geomagnetic field have been proposed for a long time. However, the first brief report of a detected change in the Earth’s magnetic field prior to an earthquake appeared only in 1964 [6]. In 1973, Tabulevich established that when the rate of change in the amplitude and period of seismic oscillations reached certain thresholds, ∂A/∂t>0.15 and ∂T/∂t>0.12, “magnetic storms” occurred unambiguously [7]. Since then, numerous publications have appeared describing the relationship between seismic and magnetic variations [8,9,10,11,12].

Following the discovery of a relationship between variations in the Earth’s magnetic field and seismic activity, new terms began to be used to describe these effects. They became known as tectonomagnetic effects [13], and the term seismogeomagnetic effect was also introduced [14]. As links between seismic events and other observable signals (electric field, telluric currents, acoustic and radio-frequency signals, etc.) were identified, such signals came to be collectively referred to as preseismic and coseismic events [15,16,17], depending on whether they preceded the earthquake (earthquake precursors) or were directly associated with the earthquake itself.

Although a strict terminology for coseismic events has not yet been fully established, they are most often classified according to the type of fields they generate. For example, seismoacoustic events [18], seismoelectromagnetic events [19], and seismoelectric and seismomagnetic events [20]. All these events, regardless of the nature of the fields they produce, can lead to variations in the magnetic field as secondary effects. The term “seismokinetic effect” is used relatively rarely [21,22]. In this work, we use this term to denote effects associated with the mechanical motion of the measuring instrument itself (the magnetometer).

The task of separating variations in different origins is quite complex. However, *only by distinguishing* these variations from one another and measuring their magnitudes can the processes occurring within the Earth during earthquakes be understood and explained [9,19]. According to [10], during strong earthquakes the observed variations range from 1.5 to 5 nT for short-period components and 2–20 nT for long-period components. At the same time, based on modeling results [19,23], the coseismic magnetic variation is estimated to be about 0.1 nT. In [24], coseismic magnetic variations in the range of 0.01–1.0 nT are considered possible under the assumption of the presence of surface charge on the Earth’s surface.

Various methods are used to separate seismomagnetic effects of different origins. For example, in [25], a characteristic feature of the seismomagnetic signal is utilized: the magnetic field vector rotates counterclockwise and exhibits circular polarization. This makes it possible, using specialized filters, to distinguish the seismomagnetic signal from the seismokinetic one, which in that work is referred to as “seismographic oscillations”.

In [26], a 4th-order Butterworth filter was applied to identify impulsive noise in magnetograms. Magnetograms from four magnetic observatories equipped with identical POS-1 scalar magnetometers were processed; for these instruments, the standard deviation value of the absolute random measurement error does not exceed 0.03 nT. The distances between the observatories ranged from 18 to 81 km. The actual standard deviation values of the four instruments were 0.11, 0.14, 0.14 and 0.28 nT. In analyzing the causes of the increased values of standard deviation of high-frequency noise, the authors suggested that the readings of the magnetometer were affected by its microseismic vibrations induced by traffic on the R43 highway, near which the “Mtb” magnetic observatory—where the maximum standard deviation value was observed—was located.

Microseismic vibrations and their causes have been studied for quite a long time. In [27], it is reported that microseismic vibrations from a water supply pump were recorded at distances of up to 5 miles. Frequencies of microseismic oscillations generated by technical equipment have been observed for up to 400 Hz [28].

The fact that seismic impacts can distort magnetograms was known earlier [29,30,31]. However, at that time it was not possible to determine the direction to the source of the seismic impact, since the magnetometers used then had a sampling rate of one measurement per minute and were unable to resolve the direction of arrival of the seismic wave. Modern magnetometers record signals at a rate of one measurement per second. Analysis of magnetograms from several nearby magnetometers employing different measurement principles has shown that events not related to seismoelectric or seismomagnetic effects may occur [32]; in that study, such variations were referred to as “instrumental error.” We do not consider this to be an “instrumental error,” but rather attribute this phenomenon to a seismokinetic event, in which oscillatory motion is experienced by the structure of the magnetic observatory itself. Under seismic excitation, this leads to oscillations of the magnetometer’s supporting structure and, consequently, to fluctuations of its local coordinate system. In [33], the phenomenon of so-called magnetic disturbances associated with the rotational motion of the coils in coil-based magnetometers was described. In [34], such rotation was modeled, and good agreement was obtained between the observed magnetic data and the amplitudes of seismic waves.

The aim of this paper is to develop a method for identifying oscillations of seismokinetic origin in magnetograms that are not related to variations in the Earth’s magnetic field itself. In the case of seismoelectric and seismomagnetic effects, the directions of the resulting electric and magnetic field vectors are related both to the directions of the ambient magnetic and electric fields and to the direction of arrival of the seismic wave. In contrast, for the seismokinetic effect, the displacement of the coordinate system should not depend on the direction of the ambient electric or magnetic fields, but only on the orientation of the local coordinate system and the direction of arrival of the seismic wave. This feature makes it possible to distinguish the seismokinetic effect from other coseismic effects. As a source of seismic waves, a seismic wave generated by a distant earthquake can be used. In the case of the seismokinetic effect, since the displacement of the coordinate system does not depend on the direction of the ambient electric and magnetic fields, these displacements can be used to determine the azimuth of arrival of the seismic impact that caused them. In turn, agreement between the estimated azimuth and the true direction to the earthquake epicenter confirms the seismokinetic nature of the observed variations. An additional indicator of the seismokinetic origin is the absence of variations in the total magnetic field vector, since changes in the projections of the vector caused by a rotation of the coordinate system should not affect its magnitude. In contrast, seismoelectric and seismomagnetic phenomena generate an additional magnetic field, which must lead to a change in the total magnetic field vector at the measurement point.

## 2. Materials and Methods

In modern magnetic observatories, the most widely used instrument for recording variations in the magnetic field is the triaxial fluxgate magnetometer. Declination–inclination magnetometers are also commonly used in combination with proton precession magnetometers or Overhauser magnetometers. Current requirements for triaxial fluxgate magnetometers specify a resolution of about 0.01 nT, while for declination–inclination magnetometers the accuracy is better than 0.1 arcminute [35]. These instruments exhibit good temperature and long-term stability. The triaxial magnetometer data used in this study were available in the XYZF format, where X, Y, and Z represent the geomagnetic field components in the geographic north, geographic east, and vertically downward directions, respectively, and F is the total magnetic field intensity.

To verify the method, magnetic field data recorded by magnetometers at the Institute of Ionosphere observatory (AAA magnetic observatory), located in Almaty, were used. The coordinates of the observatory are 43.176° N 76.952° E, at an elevation of 1266 m above sea level. Magnetic field measurements were carried out using a variational station based on the LEMI-018 magnetometer (Lviv, Ukraine) with an adjustable support of sensor unit [36]. The main technical specifications of the magnetometer are presented in Table 1. The measurements were performed at a sampling rate of 1 sample per second.

In the case of the seismokinetic effect, the azimuth calculated from the coordinate displacements should coincide with the true azimuth to the earthquake epicenter, thereby confirming the seismokinetic nature of the observed variations.

**Figure 1:**
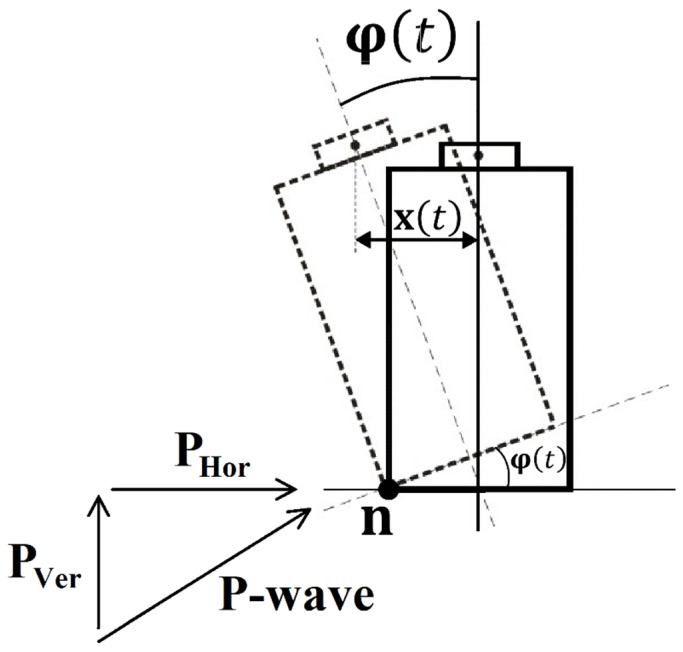
Model of the magnetometer support tilt.

The first seismic wave to reach the observation point is the P-wave, which propagates through the volume of rock [38]. When a seismic wave arrives at the magnetometer at an angle to the Earth’s surface, the vertical component of the dynamic impact causes vertical displacement of the magnetometer without altering its geographic orientation. Only the horizontal component of the seismic impact leads to tilting of the magnetometer, additionally causing horizontal displacement. Therefore, only the horizontal component of the dynamic impact is considered in the equations. When a seismic wave arrives, the magnetic field sensor experiences a displacement x(t) and a tilt φ(t) (Figure 1). The model of the magnetometer support tilt shown in Figure 1 is based on the model presented in [39], which adequately describes oscillations of the magnetometer support during earthquakes. In this model, the support subjected to the base displacement x(t) is represented as a parallelepiped that tilts by an angle φ toward the direction from which the seismic wave arrives. The axis of rotation n lies on the Earth’s surface and is parallel to the seismic wavefront.

The rotation of the magnetic field sensor (magnetometer) is described by the vector (1):

(1) φ=φn

where φ is the rotation angle in radians; n is a unit vector directed along the axis of rotation.

If the magnetometer tilts, the projections of the Earth’s magnetic field vector B onto the axes of the local Cartesian coordinate system of the magnetometer change as a result of the sensor rotation. The transformed vector $B^{\prime }$ depends on the rotation vector φ. For magnetic observatories that satisfy the requirements of [40] a small spatial displacement of the magnetometer coordinates x(t) (on the order of centimeters) cannot lead to a change in the magnitude of the magnetic field vector; therefore, it is sufficient to consider only the rotation of the coordinate system about the vector n passing through the origin of the magnetometer’s coordinate system.

**Figure 2:**
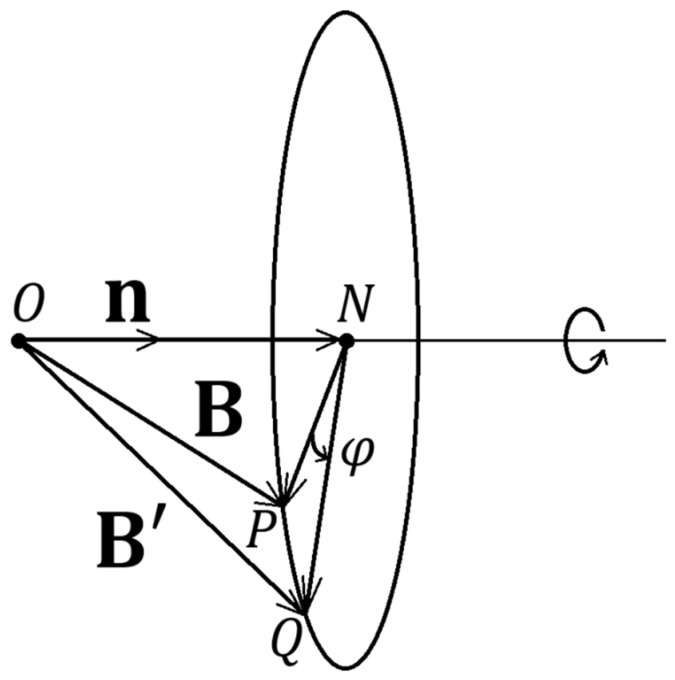
Rotation of a vector about the axis n.

The transformation of Cartesian coordinate system is illustrated in Figure 2.

**Figure 3:**
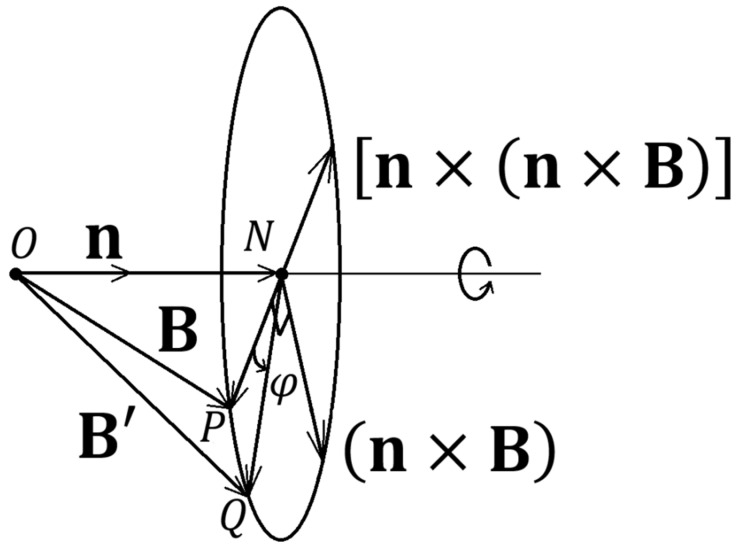
Illustration of coordinate transformation.

When the vector B rotates about the vector n, it traces a cone around the axis of rotation. The initial position of the vector B is denoted in Figure 3 as $\vec{OP}$, and the final position of the vector $B^{\prime }$ as $\vec{OQ}$. The projection of $\vec{OP}$ onto the base plane of the rotation cone is denoted as $\vec{NP}$, and the projection of $\vec{OQ}$ as $\vec{NQ}$. The lengths of the vectors $\vec{NP}$ and $\vec{NQ}$ are equal, and the angle between them is equal to φ. To establish the relationship between the vectors B and $B^{\prime }$, we represent the vector $B^{\prime }$ as the sum of two vectors (2):

(2) $$B^{\prime }=\vec{ON}+\vec{NQ}$$

In Figure 3, which illustrates the vector transformations, the vector $\vec{NQ}$ lies in the same plane as the mutually orthogonal vectors $\vec{NP}$ and (n×B), both having the same magnitude as $\vec{NQ}$, but $\vec{NQ}$ is rotated by an angle φ.

The vector $\vec{NP}$, in turn, can be expressed mathematically as [n×(n×B)]. Accordingly, the vector $\vec{NQ}$ can be described by Equation (3) as the sum of its projections onto two orthogonal vectors:

(3) $$\vec{NQ}=-\cos\varphi \left[n\times \left(n\times B\right)\right]+\sin\varphi \left(n\times B\right)$$

The vector $\vec{ON}$ can also be expressed as the sum of two vectors (4):

(4) $$\vec{ON}=B+n\times \left(n\times B\right)$$

Thus, the Earth’s magnetic field vector B, under rotation of the coordinate system, is transformed into the vector $B^{\prime }$:

(5) $$B^{\prime }=\vec{ON}+\vec{NQ}=B+n\times \left(n\times B\right)-\cos\varphi \left[n\times \left(n\times B\right)\right]+\sin\varphi \left(n\times B\right)$$

However, the rotation vector φ cannot be ***uniquely*** determined from the known vectors B and $B^{\prime }$, since an additional arbitrary rotation of the coordinate system about the vector $B^{\prime }$ does not change the result of the overall transformation. This ambiguity can be removed by supplementing the problem with the information that the rotation vector φ, as follows from Equation (1), directed along the axis of rotation, will lie only in the horizontal plane XOY of the magnetic observatory coordinate system. This eliminates the ambiguity in solving the problem. During an earthquake, the magnetic field sensor undergoes only small angular rotations. For small angles of rotation, sinφ≪1; therefore one can assume sinφ≈φ and cosφ≈1. Thus, Equation (5) can be simplified to expression (6):

(6) $${∆B=B}^{\prime }-B=\varphi \left(n\times B\right)=\varphi \times B$$

In coordinate form, Equation (6) takes the form of the system of Equation (7):

(7) $$\begin{array}{c} ∆B_{x}=\varphi_{y}B_{z}-\varphi_{z}B_{y}\\ ∆B_{y}={\varphi_{z}B_{x}-\varphi }_{x}B_{z}\\ ∆B_{z}=\varphi_{x}B_{y}-\varphi_{y}B_{x} \end{array}$$

The additional condition that the rotation vector φ lies in the horizontal plane XOY can be expressed by Equation (8):

(8) $$n_{z}=0$$

Thus, the system of Equation (7) can be simplified to the form (9):

(9) $$\begin{array}{c} ∆B_{x}=\varphi_{y}B_{z}\\ ∆B_{y}={-\varphi }_{x}B_{z}\\ ∆B_{z}=\varphi_{x}B_{y}-\varphi_{y}B_{x} \end{array}$$

From the first two equations of system (9), the rotation vector φ can be expressed in terms of the magnetic field increments (10):

(10) $$\begin{array}{c} \varphi_{x}=-\frac{∆B_{y}}{B_{z}}\\ \varphi_{y}=\frac{∆B_{x}}{B_{z}}\\ \varphi_{z}=0 \end{array}$$

The third equation in (9), which describes the relationship between the increments of the magnetic field components for rotations about an axis lying in the horizontal plane, can be transformed using Equation (10) into the form (11):

(11) $$∆B_{z}=-\left(\frac{∆B_{y}B_{y}}{B_{z}}+\frac{∆B_{x}B_{x}}{B_{z}}\right)$$

**Figure 4:**
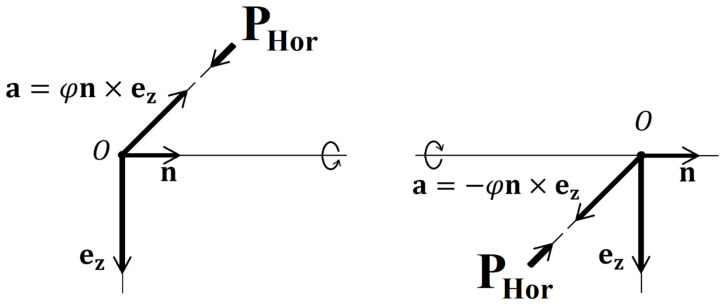
The vector a indicating the direction to the earthquake epicenter.

Since the seismic wave arrives from a direction perpendicular to the axis of rotation, which is oriented along the wavefront, the vector a indicating the direction to the earthquake epicenter is given by the cross product of the rotation vector φ and the vertical unit vector $e_{z}$ (Figure 4), directed along the OZ axis (to the center of the mass of the Earth) of the magnetic observatory coordinate system (12):

(12) $$a=\varphi \times e_{z}$$

As a result, the direction to the earthquake epicenter can be determined from the changes in the magnetic field components using Equation (13):

(13) $$\begin{array}{c} a_{x}=\frac{∆B_{x}}{B_{z}}\\ a_{y}=\frac{∆B_{y}}{B_{z}}\\ a_{z}=0 \end{array}$$

Another indication of the correct identification of the seismokinetic nature of variations in magnetograms is the absence of variations in the magnetic field when it is measured by a magnetometer that records the total field of the Earth. Rotation of the magnetometer’s coordinate system should not lead to changes in the total field itself.

## 3. Results

For verification of the method, two earthquakes with noticeable felt intensity in the city of Almaty, Kazakhstan, and one earthquake in the United States were selected.

The first two earthquakes affected the magnetic field recordings made by the magnetometers of the Institute of Ionosphere Observatory (AAA magnetic observatory) located in Almaty.

The first analyzed event was associated with an earthquake that occurred on 22 January 2024, at 18:09:04 (UTC), located 128 km WNW of Aykol (China). According to USGS data [41], the earthquake had a moment magnitude mw 7.0, with epicenter coordinates of 41.256° N 78.654° E, an elevation of 3513 m above sea level (Google Earth), and a hypocentral depth of 13.0 km.

**Figure 5:**
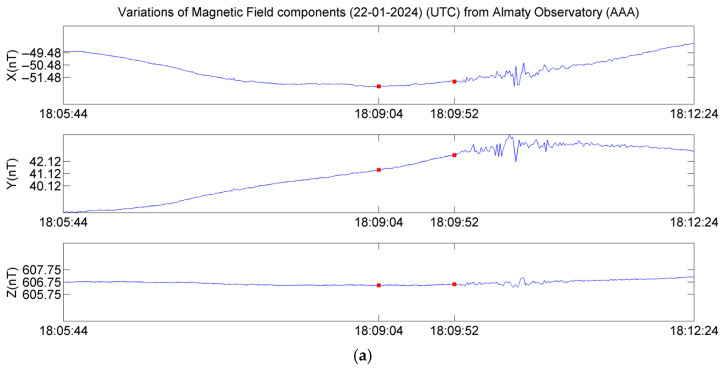

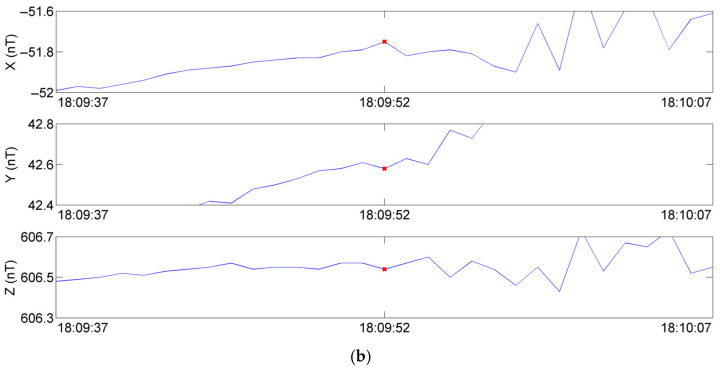
(**a**) Time of earthquake onset at the epicenter: 18:09:04; onset time of noticeable oscillations in the magnetogram components: 18:09:52; (**b**) expanded view of the onset of oscillation. The red marker indicates the reference value used to calculate $∆B_{x}$ and $∆B_{y}$.

Figure 5 shows oscillations of the magnetogram components recorded at the AAA magnetic observatory (22 January 2024). Red markers indicate the time of the earthquake at the epicenter and the onset time of noticeable oscillations in the magnetogram components. The onset time of these oscillations was determined by a sharp increase (jump) in the time derivative of the magnetograms using the method developed in [26].

The time difference between the earthquake origin and the onset of noticeable oscillations in the magnetogram components is 48 s. The distance between the earthquake epicenter and the AAA magnetic observatory is 256 km. Since an acoustic–gravity wave, due to its low propagation speed, cannot reach the ionosphere within 48 s and thus cannot cause coseismic magnetic disturbances [42,43], the recorded coseismic event can be associated with the arrival of the seismic wave at the location of the magnetic observatory or with local magnetic interference from a nearby source. Because the probability that all magnetic observatories considered in this study simultaneously recorded local magnetic interference at the time of the seismic-wave arrival is extremely low, only the seismokinetic origin of the observed event is considered in the following analysis.

**Figure 6:**
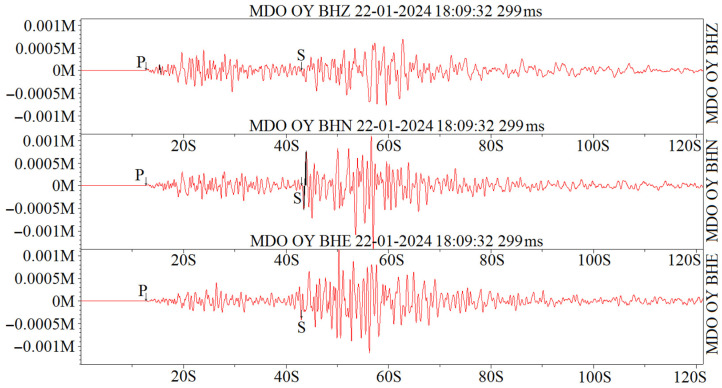
Seismogram of the 22 January 2024 (UTC) earthquake. The seismogram was provided by the Institute of Seismology (Almaty).

Figure 6 shows the seismogram of the 22 January 2024 earthquake recorded by the Medeo (MDO) seismic station, located at 43.164° N 77.047° E.

The coordinates of the magnetometer and the seismometer are separated by a distance of about 8 km. As can be seen from the magnetogram (Figure 5), noticeable signal variations began to appear at 18:09:52 UTC, whereas the seismogram (Figure 6) shows the onset of seismic oscillations at 18:09:50 UTC. The difference in the arrival time of the seismic impact at the magnetic and seismic observatories does not exceed 2 s. The duration of the seismic impact, both in the magnetogram and in the seismogram, is about 1 min. As follows from the seismogram, the seismic oscillations are broadband, with most of the energy concentrated in the frequency range of a few hertz. A magnetometer with a sampling rate of 1 Hz cannot capture oscillations at such frequencies; however, spectral analysis of the magnetograms revealed the presence of oscillations at frequencies of 0.1 Hz and higher. Since the frequency of the seismic oscillations exceeds the sampling rate of the magnetometer, aliasing prevents the magnetometer signal variations from matching the seismic oscillations exactly. However, the arrival front of the seismic wave can still be identified.

We verified the seismokinetic origin of the magnetic variations shown in the magnetograms in Figure 5. For this purpose, a comparison was made between measurements obtained from an absolute magnetometer and a variational magnetometer located in nearby pavilions. Given a seismic-wave velocity of several kilometers per second, the distance between the pavilions has a negligible effect on the recorded arrival time of the wavefront at a sampling interval of 1 s.

The total magnetic field was reconstructed from the components of magnetic variations with the addition of baseline values. The reconstructed total field was then compared with the total magnetic field measured by an absolute magnetometer POS-1 (Table 2).

**Figure 7:**
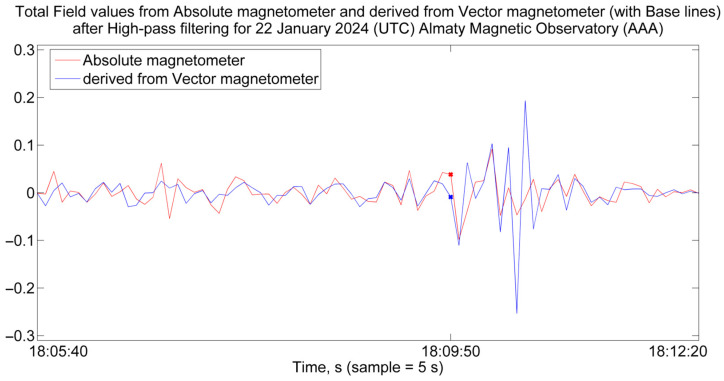
The red and blue markers indicate the onset time of noticeable oscillations in the magnetogram components, i.e., the moment of the main arrival of the seismic wave at the observatory.

The highest sampling rate available for the absolute magnetometer in 2024 was 1/5 Hz. Therefore, the per second values of the total field reconstructed from the variational magnetometer components with per second values were averaged to a frequency of 1/5 Hz. The matching of the two graphs is shown in Figure 7. The total field signals were processed using a 4th-order Butterworth high-pass filter to remove low-frequency components.

**Figure 8:**
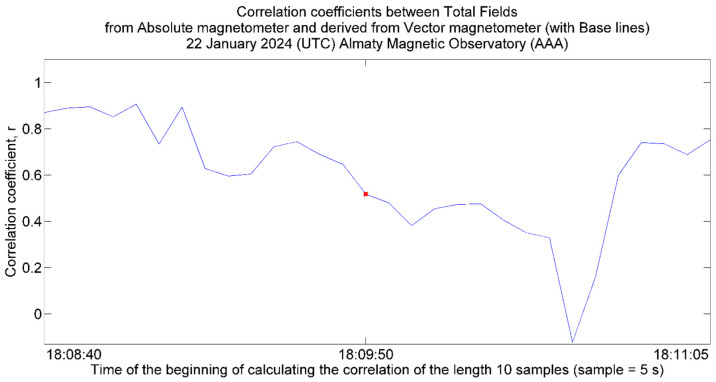
The sliding correlation window is 10 samples (1 sample corresponds to 5 s). The red marker indicates the onset time of noticeable oscillations in the magnetogram components, i.e., the moment of the main arrival of the seismic wave at the observatory.

From Figure 7, it can be observed that during the time interval of the earthquake, the total magnetic field reconstructed from the variometer components (blue curve) differs significantly from the total field measured by the absolute magnetometer (red curve). For quantitative assessment, a correlation analysis of the two magnetograms was performed using a sliding window of 10 samples (one sample corresponds to 5 s). The result of the correlation analysis is presented in Figure 8.

Figure 8 shows that during the earthquake interval, the correlation between the magnetometer readings decreased from 0.9 to 0. Seismoelectric and seismomagnetic manifestations of an earthquake would affect the measurements of both magnetometers simultaneously, and the correlation would be preserved. In the case of the seismokinetic effect, the readings of the variational magnetometer depend on changes in its orientation. When a vector magnetometer is subjected to high-frequency seismic vibrations whose frequencies exceed the magnetometer sampling rate, accurate reconstruction of the total magnetic field from the vector magnetometer measurements becomes impossible. Due to aliasing caused by the low data sampling rate, the spectral structure of the reconstructed signal is modified, which leads to a loss of correlation with the measurements of the absolute magnetometer.

The second analyzed event was associated with an earthquake that occurred on 4 March 2024, at 06:22:07 (UTC), located 25 km NW of Cholpon-Ata (Kyrgyzstan). According to USGS data [44], the earthquake had a moment magnitude mw 4.7, with epicenter coordinates of 42.836° N 76.897° E, an elevation of 3286 m above sea level (Google Earth), and a hypocentral depth of 28.3 km.

**Figure 9:**
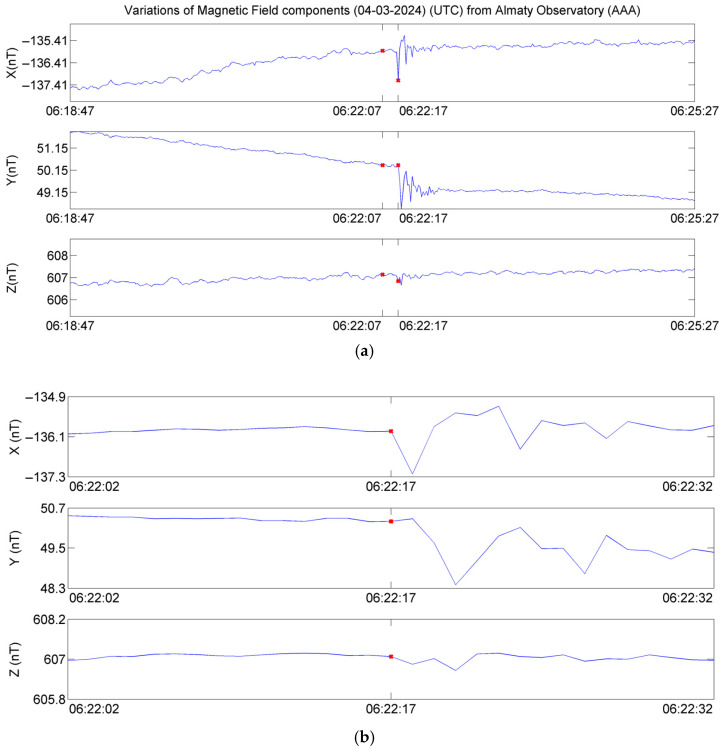
(**a**) Time of earthquake onset at the epicenter: 06:22:07; onset time of noticeable oscillations in the magnetogram components: 06:22:17; (**b**) Expanded view of the onset of oscillation. The red marker indicates the reference value used to calculate $∆B_{x}$ and $∆B_{y}$.

Figure 9 shows oscillations of the magnetogram component records registered at the AAA magnetic observatory (4 March 2024). Red markers indicate the time of the earthquake at the epicenter and the onset time of noticeable oscillations in the magnetogram components.

The time difference between the earthquake and the onset of noticeable oscillations in the magnetogram components was 10 s. The distance between the earthquake epicenter and the AAA magnetic observatory was 38 km.

We also verified the seismokinetic origin of the magnetic variations observed in the magnetograms in Figure 9.

**Figure 10:**
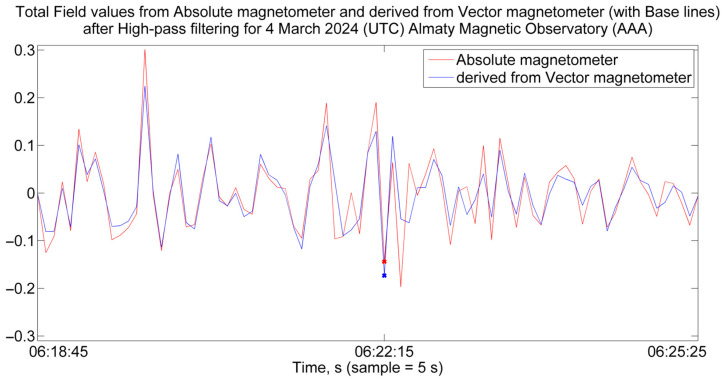
The red and blue markers indicate the onset time of noticeable oscillations in the magnetogram components, i.e., the moment of the main arrival of the seismic wave at the observatory.

The comparison of the total field curves obtained from the absolute magnetometer and reconstructed from the variational data is presented in Figure 10. The total fields were processed using a 4th-order Butterworth high-pass filter to remove low-frequency components.

**Figure 11:**
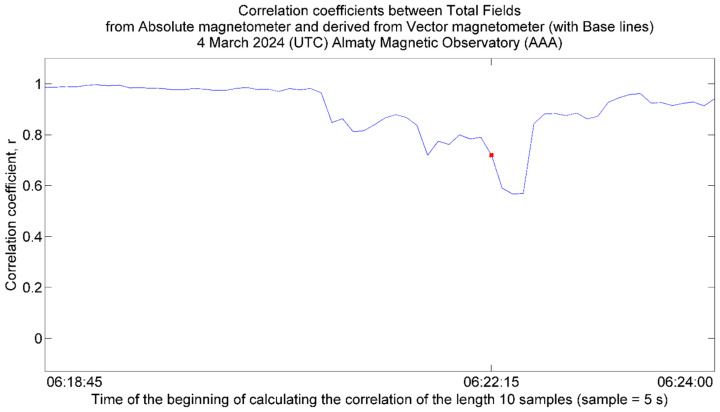
The sliding correlation window is 10 samples (1 sample corresponds to 5 s). The red marker indicates the onset time of noticeable oscillations in the magnetogram components, i.e., the moment of the main arrival of the seismic wave at the observatory.

From Figure 10, it can be observed that during the earthquake interval, the total magnetic field reconstructed from the variometer components (blue curve) also differs significantly from the total field measured by the absolute magnetometer (red curve). For quantitative assessment, a correlation analysis of the two magnetograms was performed using a sliding window of 10 samples (one sample corresponds to 5 s). The result of the correlation analysis is presented in Figure 11.

From Figure 11, it can be observed that during the earthquake interval, the correlation decreased from 0.98 to 0.57. Seismoelectric and seismomagnetic manifestations of an earthquake would affect the measurements of both magnetometers simultaneously, and the correlation would have been preserved.

From Figure 7 and Figure 10, it can be seen that although a violation of the correlation coefficient is indicative of the presence of a seismokinetic effect, seismoelectric and seismomagnetic manifestations of the earthquake may also be present. However, this cannot be determined without corrected variometer data. This issue is discussed in the Discussion section.

The third analyzed event was associated with an earthquake that occurred on 6 July 2019, at 03:19:53 (UTC) in Ridgecrest (USA). According to USGS data [45], the earthquake had a moment magnitude mw 7.1, with epicenter coordinates of 35.770° N 117.599° W, an elevation of 675 m above sea level (Google Earth), and a hypocentral depth of 8.0 km.

Magnetograms from the Fresno (FRN), Tucson (TUC) and Boulder (BOU) observatories in the USA, which were affected by this earthquake, were analyzed. INTERMAGNET database provides magnetograms for this day of both the components and the total magnetic field recorded at the FRN, TUC and BOU observatories with a sampling rate of 1 Hz. Unfortunately, neither the observatory websites [46,47,48] nor the INTERMAGNET website [49] specify the models of the vector magnetometers used for measurements. Indirect evidence suggests that Narod-type magnetometers are employed, with magnetic sensors suspended from the upper cover of the measurement unit by a gimbal mount. Such a suspension should make the magnetometer insensitive to small tilts relative to the vertical axis.

The coordinates of the Fresno (FRN) magnetic observatory are 37.090° N 119.72° W, at an elevation of 331 m above sea level.

**Figure 12:**
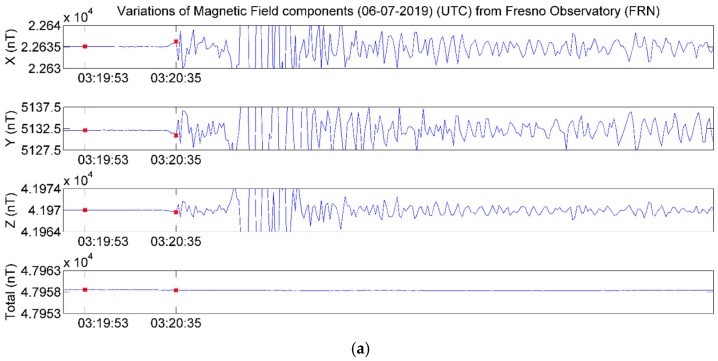

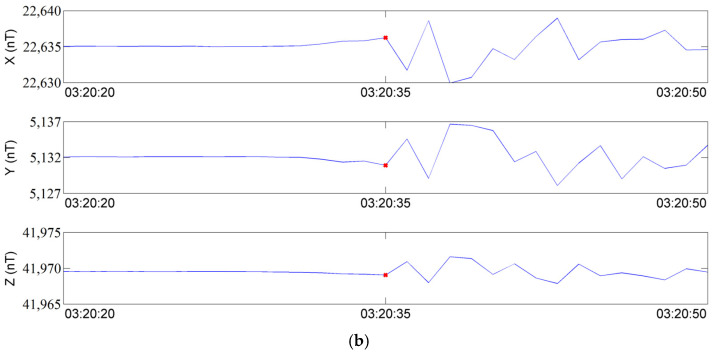
(**a**) Time of earthquake onset at the epicenter: 03:19:53; onset time of noticeable oscillations in the magnetogram components: 03:20:35; (**b**) Expanded view of the onset of oscillation. The red marker indicates the reference value used to calculate $∆B_{x}$ and $∆B_{y}$.

Figure 12 shows oscillations of the magnetogram components of the magnetic field recorded at the FRN magnetic observatory with a sampling rate of 1 Hz (6 July 2019). The total field magnetogram from the INTERMAGNET database for the FRN observatory, available at a 1 Hz sampling rate, is also shown. Red markers indicate the time of the earthquake at the epicenter and the onset time of noticeable oscillations in the magnetogram components. The onset time of noticeable oscillations was determined by a sharp increase in the time derivative of the magnetograms using the method developed in [26].

The time difference between the earthquake and the onset of noticeable oscillations in the magnetogram components was 42 s. The distance between the earthquake epicenter and the FRN magnetic observatory was 240.05 km.

The coordinates of the Tucson (TUC) magnetic observatory are 32.170° N 110.73° W, at an elevation of 946 m above sea level.

**Figure 13:**
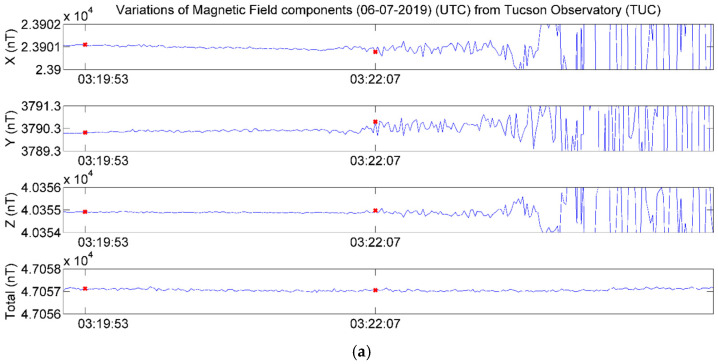

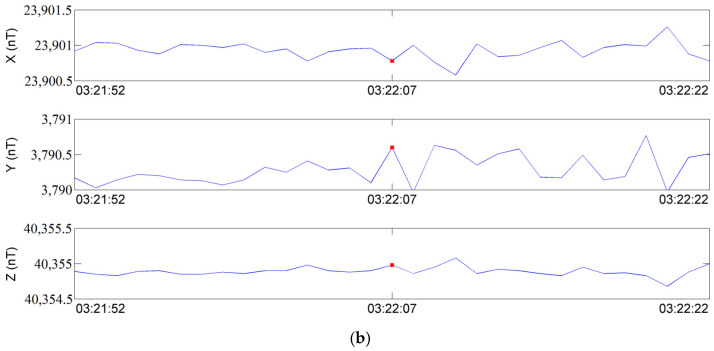
(**a**) Time of earthquake onset at the epicenter: 03:19:53; onset time of noticeable oscillations in the magnetogram components: 03:22:07; (**b**) expanded view of the onset of oscillation. The red marker indicates the reference value used to calculate $∆B_{x}$ and $∆B_{y}$.

Figure 13 shows oscillations of the magnetogram components of the magnetic field recorded at the TUC magnetic observatory with a sampling rate of 1 Hz (6 July 2019). The total field magnetogram from the INTERMAGNET database for the TUC observatory, available at a 1 Hz sampling rate, is also shown. Red markers indicate the time of the earthquake at the epicenter and the onset time of noticeable oscillations in the magnetogram components. The onset time of noticeable oscillations was determined by a sharp increase in the time derivative of the magnetograms using the method developed in [26].

The time difference between the earthquake and the onset of noticeable oscillations in the magnetogram components was 134 s. The distance between the earthquake epicenter and the TUC magnetic observatory was 749.65 km.

The coordinates of the Boulder (BOU) magnetic observatory are 40.140° N 105.233° W, at an elevation of 1682 m above sea level.

**Figure 14:**
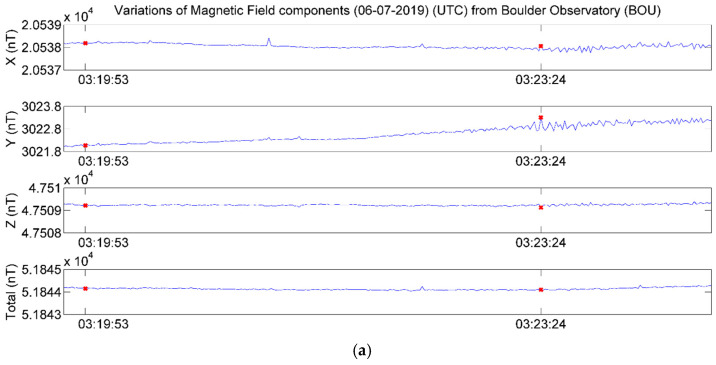

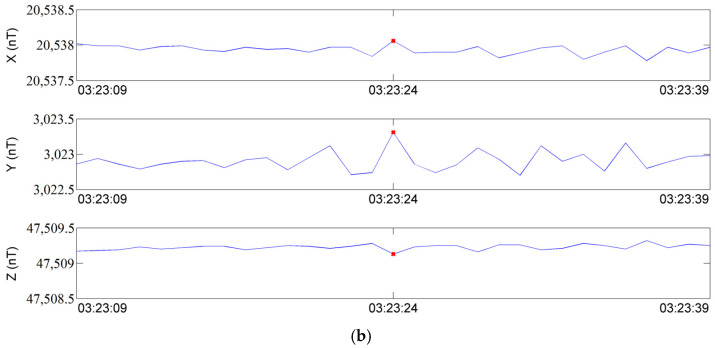
(**a**) Time of earthquake onset at the epicenter: 03:19:53; onset time of noticeable oscillations in the magnetogram components: 03:23:24; (**b**) expanded view of the onset of oscillation. The red marker indicates the reference value used to calculate $∆B_{x}$ and $∆B_{y}$.

Figure 14 shows oscillations of the magnetogram components of the magnetic field recorded at the BOU magnetic observatory with a sampling rate of 1 Hz (6 July 2019). The total field magnetogram from the INTERMAGNET database for the BOU observatory, available at a 1 Hz sampling rate, is also shown. Red markers indicate the time of the earthquake at the epicenter and the onset time of noticeable oscillations in the magnetogram components. The onset time of noticeable oscillations was determined by a sharp increase in the time derivative of the magnetograms using the method developed in [26].

The time difference between the earthquake and the onset of noticeable oscillations in the magnetogram components was 211 s. The distance between the earthquake epicenter and the BOU magnetic observatory was 1188.72 km.

The availability of total magnetic field magnetograms recorded at a sampling rate of 1 Hz by the FRN, TUC, and BOU observatories makes it possible to compare them directly with the component magnetograms without the need for reconstruction. Seismoelectric and seismomagnetic manifestations of an earthquake would be expected to appear simultaneously in both. Measurements of the total field are not affected by the tilting of the absolute magnetometer, whereas variational magnetometers are sensitive to the orientation of their components.

A second confirmation of the seismokinetic origin is the ability to reconstruct the azimuth of arrival of the seismic impact from the component values of the variometer. Agreement with the azimuth determined from seismic station data confirms the seismokinetic nature of the observed variations. To recover the azimuth of the seismic impact from changes in the magnetic field components, expression (14), derived from the developed method, was applied:

(14) $$\alpha =arctg\frac{a_{y}}{a_{x}}$$

The performance of the method for determining the azimuth toward the earthquake epicenter was validated using three earthquakes across five magnetic observatories. The azimuth calculations were performed in the MATLAB R2012a environment. The implementation code is available from the corresponding author upon request. For all events, the azimuths toward the earthquake epicenters, measured clockwise from the north at the locations of the magnetic observatories, and their deviations from the azimuths calculated using USGS data are presented in Table 3.

**Figure 15:**
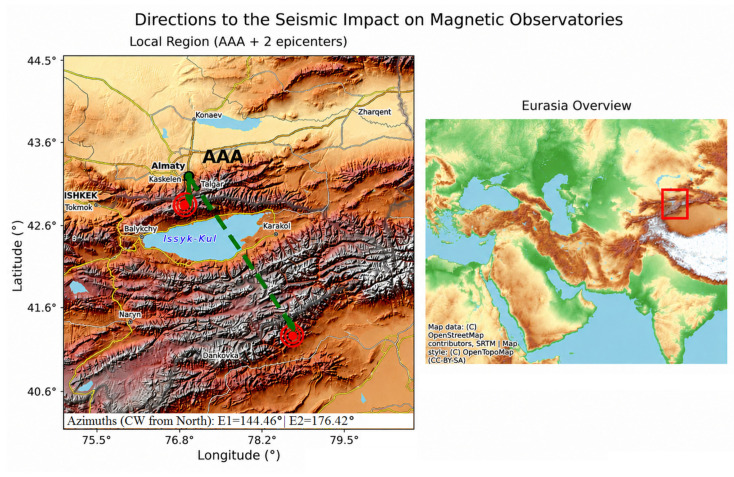
Azimuths of the directions to the epicenters of the 22 January 2024 and 4 March 2024 earthquakes relative to the AAA magnetic observatory in Almaty. The inset map on the right shows the location of the study area within Eurasia.

On the left side of Figure 15, the azimuths determined from the AAA magnetic observatory data are shown for the earthquake of 22 January 2024, located 128 km WNW of Aykol (China), and for the earthquake of 4 March 2024, located 25 km NW of Cholpon-Ata (Kyrgyzstan). The right panel shows the location of the study area within the Eurasian region.

**Figure 16:**
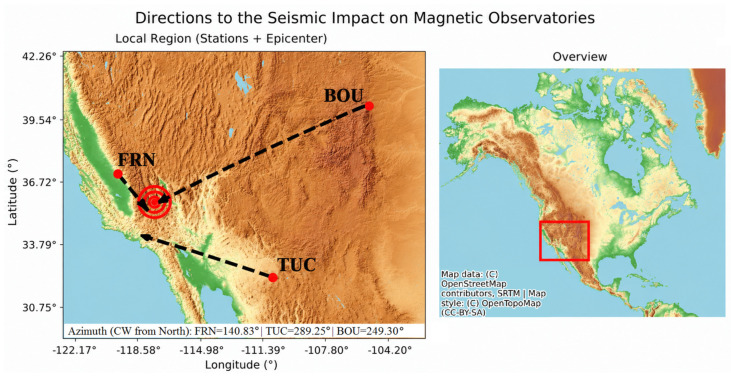
Azimuths of the directions to the epicenter of the 6 July 2019 earthquake relative to the FRN, TUC and BOU magnetic observatories in the USA. The inset map on the right shows the location of the study area within North America.

On the left side of Figure 16, the azimuths determined from data from the FRN, TUC, and BOU magnetic observatories are shown for the 6 July 2019 earthquake at Ridgecrest (USA). The right panel shows the location of the study area within North America.

## 4. Discussion

Noting the discrepancy between the readings of the vector and absolute magnetometers, we attempted to separate seismokinetic events from coseismic events of other origins, whose magnitudes—according to calculations by many authors—should be one to two orders of magnitude smaller than the values typically recorded by the vector magnetometer. We hypothesized that if the oscillations associated with mechanical vibrations of the magnetometer support are removed from the vector magnetometer readings, the remaining variations would be related to seismomagnetic and seismoelectric effects. Subsequently, the seismomagnetic variations could be separated using a polarization filter, as described in [25].

In addition, based on data from the AAA observatory, it was found that regardless of the azimuth from which the seismic wave arrived, only the signal variations corresponding to the first seismic impact provided the correct azimuth of arrival. Subsequently, the magnetometer began to oscillate in similar directions. Most likely, these were resonant oscillations of the magnetometer support, the direction of which may be related to features of its construction. Thus, the identification of seismoelectric and seismomagnetic variations proved to be impossible without additional data on the oscillations of the magnetometer support, since interference caused by tilts of the magnetometer due to seismic vibrations exceeds the levels of the actual seismomagnetic and seismoelectric signals. To eliminate this interference, it is necessary either to reduce the level of microseismic signals in the magnetic observatory or to correct the results of vector magnetometer measurements associated with micro-oscillations of the instrument.

INTERMAGNET requires that the accuracy of measurements of the Earth’s magnetic field be no worse than 0.1 nT, although many magnetic observatories are equipped with more advanced magnetometers with an accuracy of 0.01 nT. For a typical level of the Earth’s static magnetic field of about 50,000 nT (typical for Kazakhstan), such magnetometers would respond to a tilt of $10^{-2}/5\times 10^{4}=2\times 10^{-7}\;rad$ or approximately 0.04 arcseconds. Such angular deviations can be measured using modern inclinometers. In [50], a precision laser inclinometer with dimensions of 20×20×20 cm and a weight of 10 kg is described, having a sensitivity of $6\times 10^{-11}\;rad/{Hz}^{1/2}$ in the frequency range $1.4\times 10^{-3}-10\;Hz$. There are also magnetometers combined with inclinometers [51]. However, they are intended to compensate for relatively slow variations in the magnetic field associated with the tilting of the magnetometers. Real seismic vibrations may have frequencies of up to 50 Hz [52]. The use of magnetometers with a digitization rate below 100 Hz (according to the Nyquist theorem requirements) for measurements of the Earth’s magnetic field leads to aliasing, and consequently makes it impossible to compensate for the tilt of the magnetometer sensor. In the present study, only the simplest model of magnetometer sensor tilting about a horizontal axis was considered. In reality, seismic vibrations may cause rotations of the sensor about multiple axes [53]. Therefore, magnetic field measurements obtained during seismokinetic events can currently only be regarded as unreliable.

As follows from the data obtained at U.S. magnetic observatories equipped with gimbal-mounted sensors, vector magnetometers respond to high-frequency seismic vibrations. It is likely that the type of gimbal suspension used in these magnetometers, due to its dynamic characteristics, is unable to respond accurately enough to rapid changes in tilt angle. It is known that when the vibration frequency approaches the resonance frequency of a gimbal suspension, measurement errors may increase by an order of magnitude. Since the proposed mathematical approach made it possible to determine the bearing from the measurement results, it can be assumed that at the moment of the seismic impact the gimbal suspension did not fully compensate for the tilt, causing the sensors to become temporarily inclined, similarly to the case of rigid mounting.

The loss of correlation between the magnetic field measurements of the absolute and vector magnetometers can be used as an indicator of data quality problems. Moreover, INTERMAGNET recommends performing cross-comparisons between vector and scalar magnetometer measurements as an effective method for supporting data quality control. We do not have a definitive explanation for this phenomenon. These discrepancies may be related to the different frequency responses of the absolute and vector magnetometers, which can lead to a reduction in the correlation coefficient when high-frequency magnetic field oscillations occur during an earthquake [12]. Also, correlation-based comparisons of the readings from absolute and vector magnetometers, which can be performed almost in real time, may serve as a reliable indicator of seismokinetic events.

## 5. Conclusions

1. A discrepancy in the total magnetic field measurements during a coseismic event, as observed between absolute and vector magnetometers, can be considered a reliable indicator of an event of seismokinetic origin.
2. Seismokinetic events have a greater impact on magnetometer readings than seismoelectric and seismomagnetic events.
3. The bearing of the direction of arrival of the seismic wave can be determined from the first seismic impact.

## Figures and Tables

**Table 1:** Specifications of the LEMI-018 magnetometer [37].

Measuring ranges of total magnetic field at the display	±68,000 nT
Resolution along each component at the display, digital output	0.01 nT
Measured range at analog output without additional compensation	±1000 nT
Transformation factor of analog output	2.4 mV/nT
Noise level at (0.03–0.3) Hz frequency band	<15 pT rms
Temperature drift	<0.2 nT/℃
Components orthogonality error	<30 min of arc
Automated offset compensation band along each component	±68,000 nT
Time of samples averaging by PC software (MF3 1.0)	1…60 s
Operating temperature range	−20 to +60 °C
Length of GPS antenna cable	3 m

**Table 2:** Specifications of the POS-1 scalar magnetometer.

Operating field range, nT	20,000–100,000
Absolute error, not exceeding, nT	1
Sensitivity (RMS), not exceeding, nT	0.03
Resolution, nT	0.001
Duration of a single measurement, not exceeding, s	3
Automatic measurement interval, s	0.5, 1, 2, 3, 4, …
Optimal sensor orientation relative to the magnetic field, deg	90±10
Gradient tolerance, nT/m	20,000
Operating temperature range, ℃	−30/+60

**Table 3:** Results of the method validation for calculating earthquake azimuths relative to the magnetic observatories, including deviations from USGS values.

№	Earthquake	Azimuth	Values of Magnetic Induction Component Changes at the Moment of First Impact (Sharp Increase in the Value)	Deviation from the Azimuth Value Calculated from USGS Data
**1**	22 January 2024, 128 km WNW of Aykol (China)	(1) Magnetic observatory AAA (256 km from epicenter, 48 s of wave propagation from epicenter)		
		144.46°	$∆B_{x}=-0.07$ nT $∆B_{y}=0.05$ nT	1.58°
**2**	4 March 2024, 25 km NW of Cholpon-Ata (Kyrgyzstan)	(1) Magnetic observatory AAA (38 km from epicenter, 10 s of wave propagation from epicenter)		
		176.42°	$∆B_{x}=-1.28$ nT $∆B_{y}=0.08$ nT	10.3°
**3**	6 July 2019, in Ridgecrest (USA)	(1) Magnetic observatory FRN (240.05 km from epicenter, 42 s of wave propagation from epicenter)		
		140.83°	$∆B_{x}=-4.53$ nT $∆B_{y}=3.69$ nT	13.86°
		(2) Magnetic observatory TUC (749.65 km from epicenter, 134 s of wave propagation from epicenter)		
		289.25°	$∆B_{x}=0.22$ nT $∆B_{y}=-0.63$ nT	14.81°
		(3) Magnetic observatory BOU (1188.72 km from epicenter, 211 s of wave propagation from epicenter)		
		249.30°	$∆B_{x}=-0.17$ nT $∆B_{y}=-0.45$ nT	0.55°

## Data Availability

The necessary data are available from the corresponding author upon reasonable request.

## References

[1] Daniell J.E., Shroder J.F., Wyss M. (2014). The Socioeconomic Impact of Earthquake Disasters. Earthquake Hazard, Risk and Disasters.

[2] Daniell J.E., Schaefer A.M., Wenzel F. (2017). Losses associated with secondary effects in earthquakes. Front. Built Environ..

[3] EMSC—Euro-Mediterranean Seismological Centre. https://www.emsc-csem.org/.

[4] Orfeus EIDA—European Integrated Data Archive. http://www.orfeus-eu.org/data/eida/.

[5] National Research Institute for Earth Science and Disaster Resilience Hi-Net—High Sensitivity Seismograph Network Japan. https://www.hinet.bosai.go.jp/?LANG=en.

[6] Masci F., Thomas J.N. (2015). Are there new findings in the search for ULF magnetic precursors to earthquakes?. J. Geophys. Res. Space Phys..

[7] Tabulevich V.N. (1973). Complex of phenomena which arise simultaneously with the generation of microseismic vibrations. Pure Appl. Geophys..

[8] Iyemori T., Kamei T., Tanaka Y., Takeda M., Hashimoto T., Araki T., Okamoto T., Watanabe K., Sumitomo N., Oshiman N. (1996). Co-Seismic Geomagnetic Variations Observed at the 1995 Hyogoken-Nanbu Earthquake. J. Geomagn. Geoelectr..

[9] Surkov V.V., Pilipenko V.A., Sinha A.K. (2018). Possible mechanisms of co-seismic electromagnetic effect. Acta Geod. Geophys..

[10] Spivak A.A., Riabova S.A. (2019). Geomagnetic Variations during Strong Earthquakes. Izv. Phys. Solid Earth.

[11] Guglielmi A., Potapov A., Tsegmed B. (2004). One mechanism for generation of the co-seismic electromagnetic oscillations. Phys. Chem. Earth Parts A/B/C.

[12] Wang Y.L., Xie T., An Y.R., Yue C., Wang J.Y., Yu C., Yao L., Lu J. (2019). Characteristics of the coseismic geomagnetic disturbances recorded during the 2008 Mw 7.9 Wenchuan Earthquake and two unexplained problems. Earth Planet. Phys..

[13] Davis P.M., Searls C.A. (1981). Magnetic field measurements in the aftershock region of the Coyote Lake earthquake. J. Geophys. Res. Solid Earth.

[14] Liu J.Y., Chen C.H., Chen Y.I., Yen H.Y., Hattori K., Yumoto K. (2006). Seismo-geomagnetic anomalies and M ≥ 5.0 earthquakes observed in Taiwan during 1988–2001. Phys. Chem. Earth Parts A/B/C.

[15] Uyeda S., Hayakawa M., Nagao T., Molchanov O., Hattori K., Orihara Y., Gotoh K., Akinaga Y., Tanaka H. (2002). Electric and magnetic phenomena observed before the volcano-seismic activity in 2000 in the Izu Island Region, Japan. Proc. Natl. Acad. Sci. USA.

[16] Mueller R.J., Johnston M.J.S. (1998). Review of magnetic field monitoring near active faults and volcanic calderas in California: 1974–1995. Phys. Earth Planet. Inter..

[17] Petko N. (2016). Recognition of unipolar magnetic field pulses as transient signals coming from the Earth’s crust. Comptes Rendus l’Académie Bulg. Des. Sci..

[18] McKee K., Fee D., Haney M., Matoza R.S., Lyons J. (2018). Infrasound signal detection and back azimuth estimation using ground-coupled airwaves on a seismo-acoustic sensor pair. J. Geophys. Res. Solid Earth.

[19] Gao Y., Chen X., Hu H., Wen J., Tang J., Fang G. (2014). Induced electromagnetic field by seismic waves in Earth’s magnetic field. J. Geophys. Res. Solid Earth.

[20] Jouniaux L., Zyserman F. (2016). A review on electrokinetically induced seismo-electrics, electro-seismics, and seismo-magnetics for Earth sciences. Solid Earth.

[21] Manglik A., Gupta S. (2025). Coseismic Electromagnetic Signals as Pseudo-Seismograms for Mapping of Upper Crustal Seismic Velocity Structure: An Example from the Ganga Basin Using Magnetotelluric Time Series of the 3 November 2023 Western Nepal Earthquake. J. Geol. Soc. India.

[22] Hallbauer-Zadorozhnaya V. (2005). Electro-kinetic soundings with a TDEM instrument. 9th SAGA Biennial Technical Meeting and Exhibition.

[23] Yamazaki K. (2012). Estimation of temporal variations in the magnetic field arising from the motional induction that accompanies seismic waves at a large distance from the epicentre. Geophys. J. Int..

[24] Ren H., Wen J., Huang Q., Chen X. (2015). Electrokinetic effect combined with surface-charge assumption: A possible generation mechanism of coseismic EM signals. Geophys. J. Int..

[25] Guglielmi A., Hayakawa H., Potapov A., Tsegmed B. (2006). Polarization method to detect the co-seismic magnetic oscillations. Phys. Chem. Earth Parts A/B/C.

[26] Vassilyev I., Mendakulov Z., Zhumabayev B., Kozin I., Beloslyudtsev O., Dossaibekova S. (2025). Improving the Quality of Magnetograms Using Data from Several Magnetic Observatories. Appl. Sci..

[27] Wilson C.D.V. (1953). An analysis of the vibrations emitted by some man-made sources of microseisms. Proc. R. Soc. London Ser. A Math. Phys. Sci..

[28] Lu B., Cheng J., Hu J., Qin S. (2011). Seismic features of vibration induced by mining machines and feasibility to be seismic sources. Procedia Earth Planet. Sci..

[29] Eleman F. (1966). The Response of Magnetic Instruments to Earthquake waves. J. Geomagn. Geoelectr..

[30] Srivastava B.J. (1967). Effects of Thunderstorms and Earthquakes on the Hyderabad Magnetographs. J. Geomagn. Geoelectr..

[31] Migunov N.I., Sobolev G.A. (2006). Seismomagnetic signals associated with Sakhalin earthquakes. Izv. Phys. Solid Earth.

[32] Masci F., Thomas J.N. (2016). Evidence of underground electric current generation during the 2009 L’Aquila earthquake: Real or instrumental?. Geophys. Res. Lett..

[33] Gao Y., Jiang P., Xu Y., Jiang L., Cheng C.-H., Zhao G., Tang J., Chen X., Han B., Chong J. (2021). On magnetic disturbances induced by rotation of coil-type magnetometer driven by seismic waves. Geophys. J. Int..

[34] Kasdi A.S., Bouzid A., Hamoudi M., Gao Y. (2022). Numerical modeling of the co-seismic electromagnetic signals observed during the 2014 Mw 4.9 Hammam Melouane earthquake, Northern Algeria. J. Appl. Geophys..

[35] Hrvoic I., Newitt L.R., Mandea M., Korte M. (2011). Instruments and Methodologies for Measurement of the Earth’s Magnetic Field. Geomagnetic Observations and Models.

[36] Type of Equipment Used. Geomagnetic Observatory “Alma-Ata”. http://geomag.ionos.kz/about.html.

[37] LEMI Sensors, The Editors of Encyclopaedia (2016). “LEMI-018”.

[38] Bolt B.A. (1978). Earthquakes: A Primer.

[39] Collini L., Garziera R., Riabova K., Munitsyna M., Tasora A. (2016). Oscillations Control of Rocking-Block-Type Buildings by the Addition of a Tuned Pendulum. Shock Vib..

[40] Jankowski J., Sucksdorff C. (1996). Guide for Magnetic Measurements and Observatory Practice.

[41] USGS Earthquake Hazards Program. M 7.0–128 km WNW of Aykol, China. https://earthquake.usgs.gov/earthquakes/eventpage/us7000lsze/executive.

[42] Gao Y., Li T., Zhou G., Chen C.-H., Sun Y.-Y., Zhang X., Liu J.-Y., Wen J., Yao C., Bai X. (2023). Acoustic-gravity waves generated by a point source on the ground in a stratified atmosphere-Earth structure. Geophys. J. Int..

[43] Zhantayev Z.S., Somsikov V.M., Andreev A.B., Zhumabayev B.T., Kapytin B.I. (2021). Optical and satellite observation of acoustic-gravity waves in the mesosphere during seismo activity. J. Open Syst. Evol. Probl..

[44] USGS Earthquake Hazards Program. M 4.7–25 km NW of Cholpon-Ata, Kyrgyzstan. https://earthquake.usgs.gov/earthquakes/eventpage/us6000mglm/executive.

[45] USGS Earthquake Hazards Program. M 7.1—Ridgecrest Earthquake Sequence. https://earthquake.usgs.gov/earthquakes/eventpage/ci38457511/origin/detail.

[46] U.S. Geological Survey (USGS) Geomagnetism Program. Fresno (FRN). https://www.usgs.gov/programs/geomagnetism/science/fresno-frn.

[47] U.S. Geological Survey (USGS) Geomagnetism Program. Tucson (TUC). https://www.usgs.gov/programs/geomagnetism/science/tucson-tuc.

[48] U.S. Geological Survey (USGS) Geomagnetism Program. Boulder (BOU). https://www.usgs.gov/programs/geomagnetism/science/boulder-bou.

[49] INTERMAGNET List of Observatories. https://intermagnet.org/metadata/imos.

[50] Atanov N.V., Bednyakov I.V., Budagov Y.A., Glagolev V.V., Klemeshov Y.V., Krasnoperov A.V., Kuzkin A.M., Lyablin M.V., Ni R.V., Pluzhnikov A.A. (2023). Compact Precision Laser Inclinometer: Measurement of Signals and Noise. Phys. Part. Nucl..

[51] Khomutov S.Y. (2017). Magnetic observations at Geophysical Observatory Paratunka IKIR FEB RAS: Tasks, possibilities and future prospects. E3S Web Conf..

[52] Schwardt M., Pilger C., Gaebler P., Hupe P., Ceranna L. (2022). Natural and Anthropogenic Sources of Seismic, Hydroacoustic, and Infrasonic Waves: Waveforms and Spectral Characteristics (and Their Applicability for Sensor Calibration). Surv. Geophys..

[53] Kurzych A.T., Jaroszewicz L.R. (2024). A Review of Rotational Seismology Area of Interest from a Recording and Rotational Sensors Point of View. Sensors.

